# Intrinsically Disordered SRC-3/AIB1 Protein Undergoes Homeostatic Nuclear Extrusion by Nuclear Budding While Ectopic Expression Induces Nucleophagy

**DOI:** 10.3390/cells8101278

**Published:** 2019-10-19

**Authors:** Miguel A. Cabrita, L. Isabel Renart, Rosanna Lau, M. A. Christine Pratt

**Affiliations:** Department of Cellular and Molecular Medicine, University of Ottawa, 451 Smyth Road, Ottawa, ON K1H 8M5, Canada

**Keywords:** transcriptional coactivator, intrinsically disordered protein, protein aggregation, nuclear protein quality control, PML, microtubules, nucleophagy

## Abstract

SRC-3/AIB1 (Amplified in Breast Cancer-1) is a nuclear receptor coactivator for the estrogen receptor in breast cancer cells. It is also an intrinsically disordered protein when not engaged with transcriptional binding partners and degraded upon transcriptional coactivation. Given the amplified expression of SRC-3 in breast cancers, the objective of this study was to determine how increasing SRC-3 protein levels are regulated in MCF-7 breast cancer cells. We found that endogenous SRC-3 was expelled from the nucleus in vesicle-like spheres under normal growth conditions suggesting that this form of nuclear exclusion of SRC-3 is a homeostatic mechanism for regulating nuclear SRC-3 protein. Only SRC-3 not associated with CREB-binding protein (CBP) was extruded from the nucleus. We found that overexpression in MCF-7 cells results in aneuploid senescence and cell death with frequent formation of nuclear aggregates which were consistently juxtaposed to perinuclear microtubules. Transfected SRC-3 was SUMOylated and caused redistribution of nuclear promyelocytic leukemia (PML) bodies and perturbation of the nuclear membrane lamin B1, hallmarks of nucleophagy. Increased SRC-3 protein-induced autophagy and resulted in SUMO-1 localization to the nuclear membrane and formation of protrusions variously containing SRC-3 and chromatin. Aspects of SRC-3 overexpression and toxicity were recapitulated following treatment with clinically relevant agents that stabilize SRC-3 in breast cancer cells. We conclude that amplified SRC-3 levels have major impacts on nuclear protein quality control pathways and may mark cancer cells for sensitivity to protein stabilizing therapeutics.

## 1. Introduction

Nuclear steroid receptor coactivator-3 (SRC-3/AIB1/NCOA3) was originally identified as a gene amplified in nearly 10% of breast cancers [1] but has been shown to be overexpressed in 50–60% of cases with weak to absent expression in normal mammary tissue [2]. SRC-3 bound to liganded nuclear receptors on chromatin recruits histone acetyltransferases (HATs) including p300/CBP and p/CAF to the complex [3]. SRC-3 coactivates nuclear receptors including the estrogen receptor (ER) [4] as well as other transcription factors [5] and is necessary for the normal development of the mammary gland [6]. While SRC-3 correlates with expression of the ER and PR in breast tumors [7] it can also be overexpressed in ER-alpha-positive (ER^+^) tumors [8].

Both the function and protein levels of SRC-3 are rigorously regulated. Multiple phosphorylations mediated by several different kinases regulate both SRC-3 function and degradation at several sites including six residues that are necessary for receptor coactivation and a subset required for NF-κB-mediated transcriptional coactivation [9]. Two other phospho-residues appear necessary to maintain function and render the protein susceptible to ubiquitin-proteosome mediated degradation [9,10]. Optimal transcriptional coactivation by SRC-3 requires phosphorylation of S505 by the GSK3 kinase and is followed by SCF^Fbw7α^-mediated mono-ubiquitination of L732 and L786 residues. As transcription proceeds SRC-3 becomes poly-ubiquitinated resulting in the turnover of SRC-3 through the 26S proteasome which is also requisite for transcription. When and why other post-translational modifications of SRC-3 occur is not understood. Removal of phosphate at S102 mediated by PP1 stabilizes the SRC-3 protein and inhibits its transcriptional activity [10]. Similarly, the transcriptional activity of SRC-3 is inhibited by SUMOylation [11,12].

A series of mid-molecule LXXLL domains facilitate SRC-3 interaction with a variety of transcription factors and a glutamine-rich CBP/p300 interaction domain is followed by a polyglutamine (polyQ) tract near the C-terminus contributing to its intrinsic disorder. The binding of SRC-3 to CBP results in a phenomenon termed “mutual synergistic helical folding” rendering a highly ordered complex [13] accompanied by a massive loss of entropy [14]. Exposed hydrophobic domains in intrinsically disordered proteins (IDPs) can interfere with protein associations, resulting in aggregation. Increases in IDPs can result in aggregated proteins that coalesce to form larger inclusion bodies or aggresomes which are associated with a variety of disease states (reviewed in [15]). Small aggregates form throughout the cytoplasm and are quickly transported toward the microtubule (MT)-organizing center, where they coalesce to form aggresomes [15,16]. Disposal of larger misfolded proteins and aggregates is not managed by the proteasome due to limitations on size for entry into the proteasome barrel and, instead, degradation occurs through autophagy [17]. However, nuclear aggregates present a challenge as the nucleus does not have access to cytoplasmic lysosomal components.

Here we demonstrate that SRC-3 nuclear protein levels are regulated in part through extrusion in vesicle-like blebs while transfection of SRC-3 results in cytotoxicity and senescence in MCF-7 cells. Overexpression of SRC-3 protein was either homogeneously distributed or in nuclear aggregates and had major ramifications on microtubules and PML nuclear bodies. We also found evidence of nucleophagy in SRC-3 overexpressing cells. Similarly, aspects of these cellular effects were observed after pharmacological stabilization of SRC-3. These data provide new insight into the rigorous regulation of nuclear SRC-3 protein levels and suggest that amplified levels of this and other intrinsically disordered transcription factors might represent a vulnerability of cancer cells to agents that stabilize these proteins.

## 2. Materials and Methods

Reagents: All chemicals were purchased from MilliporeSigma Canada (Oakville, Canada) unless otherwise stated. Bortezomib was purchased from (AdooQ Bioscience, Irvina, CA, USA).

Cell culture: MCF-7 and LCC9 cells were the generous gift of Dr. Robert Clarke (Georgetown University, Washington, DC, USA). MCF-10A cells were obtained from the ATCC. All cells were cultured according to ATCC guidelines. MCF-7 cells stably expressing GFP-LC-3 were a generous gift of Dr. Sharon Gorski (Simon Fraser University, Burnaby, BC, Canada) [18]. In some experiments, cells were treated at approximately 50% confluence with hydroxychloroquine (HCQ) (50 µM) or bortezomib (B) (25 nM) for 48 h prior to harvest.

Site-directed Mutagenesis: pCMV-Flag-SRC3 (a gift from Dr. B.W. O’Malley, Baylor College of Medicine, Houston, TX, USA) was used as the DNA template to make two modifications to the SRC3 cDNA. The Flag-SRC3 poly-glutamine stretch deletion (polyQdel) mutant (lacking 29 glutamine residues (amino acids 1248–1276) was generated using the QuikChange II Site-directed Mutagenesis Kit (Agilent, Mississauga, ON, Canada) according to the manufacturer’s instructions. The Flag-SRC3-S102A mutant was produced using the Q5 Site-directed Mutagenesis Kit (New England Biolabs, Whitby, ON, Canada) according to the enclosed instructions.

Transfections: Transient Transfections: MCF-7 cells were transiently transfected using either polyethylenimine, Fugene 6 (Roche, Laval, QC, Canada) or Viafect transfection reagent (Promega, Madison, WI, USA) and lysates harvested or immunofluorescence performed 72 h post-transfection. Plasmids were pcDNA3, pcDNA3-RFP (a gift from Dr. B. Tuana, University of Ottawa, Ottawa, ON, Canada), pEGFP-SRC3, pEGFP-T24A, pEGFP-SRC3S860A, pEGFP-SRC3-S505A and pEGFP-SRC3-S509A (generous gifts from Dr. B.W. O’Malley) [10]. The NLS-GFP^u^ plasmid was a gift from Dr. R.R. Kopito (Stanford University, Stanford, CA, USA) [19].

Generation of Stable Cell lines: MCF-7 cells were co-transfected with either pcDNA3 or pCMX.F.RAC3 (SRC-3) (gift of Dr. Don Chen, Rutgers University, Piscataway, NJ [20]) with pCMV-puromycin. 48 h post-transfection, cells were grown in 4 µg/mL puromycin for 2–4 weeks. Colonies were isolated individually, categorized according to phenotype and expanded prior to assay for protein expression by immunoblot analysis. Typically, clones were passaged 3 times prior to analyses. Established clones were maintained in 2 µg/mL puromycin.

Antibodies: The following primary antibodies were used in this study for immunoblot analysis and/or indirect immunofluorescence: anti-AIB1 mouse IgGl #611105 (BD Bioscience, Transduction Laboratories, San Jose, CA, USA), rabbit anti-GFP(#G1544), anti-tubulin-FITC (clone DM1A), rabbit anti-actin antibody (#A2066) (MilliporeSigma), rabbit anti-CBP(sc-369), anti-p21(sc-397) (Santa Cruz, Dallas, TX), anti-lamin B1(ab16048), rabbit-anti-cyclin E1(ab7959), rabbit anti-vinculin(ab129002), anti-Chk2(ab47433) (Abcam, Cambridge, MA), anti-PML(#PA5-80909), anti-SUMO-1(#PA5-11312) and anti-RanGAP1(#33-0800) (ThermoFisher Scientific/Invitrogen, Ottawa, ON, Canada), anti-phospho Chk2(Thr68)(#2661S), rabbit anti-PARP-1(#9542) (Cell Signaling Technology/NEB, Whitby, ON, Canada), anti-LC3(NB100-2220) (Novus Biologicals/BioTechne, Oakville, ON, Canada). The following secondary antibodies were used for immunoblot analysis or immunocytochemistry: peroxidase-conjugated AffiniPuro goat anti-rabbit IgG (H + L), peroxidase-conjugated AffiniPuro goat anti-mouse IgG (H + L), FITC-conjugated peroxidase-conjugated AffiniPuro goat anti-rabbit IgG (H + L), Cy3-conjugated AffiniPuro goat antimouse IgG (H + L) (all JacksonImmunoResearch Laboratories, Inc., West Grove, PA), goat anti-rabbit Alexa 488, goat anti-mouse Alexa 555 (ThermoFisher/Invitrogen).

SDS-polyacrylamide gel electrophoresis (SDS-PAGE) and immunoblotting: A modified, highly porous discontinuous glycerol-containing SDS-PAGE [21] was used and immunoblotting was performed as previously described [22]. Typically, 20–25 µg of protein was loaded per lane.

Immunoprecipitation: MCF-7 cells were transfected with pCMV-FLAG-SRC-3 wt or the empty vector, pcDNA3. After 36 h, cells were lysed on ice in RIPA buffer containing proteasome and phosphatase inhibitors. Lysates were cleared by centrifugation, then 500 µg of protein were incubated with either 1 µg of anti-AIB1 (SRC3) monoclonal Ab (BD Bioscience) or 1 µg of mouse control IgG (Jackson ImmunoResearch) overnight with continuous rotation at 4 °C. 20 µL Protein A/G PLUS agarose beads (Santa Cruz) were added for 1 h at 4 °C. Immunoprecipitates were collected by centrifugation (1000× *g*, 5 min, 4 °C) and washed pellets were resuspended in 20 µL of 2× SDS-PAGE loading buffer, boiled and centrifuged prior to loading on an SDS-PAGE gel for immunoblotting.

Viral Transduction: MCF-7 cells were transduced with one of the following adenoviruses (Ad-SRC3, Ad-LacZ or Ad-RFP) at the multiplicity of infection (M.O.I.) of 10. The adenovirus expressing SRC3 was a gift from Dr. H.W. Chen (UC Davis, Sacramento, CA, USA)) [23] and the adenovirus expressing RFP was a gift from Dr. Robin Parks (Ottawa Hospital Research Institute, Ottawa, ON, Canada). Briefly, cells were plated in media in the absence of serum. After 45 min, adenovirus was added to the cells and 2.5 h later, an equal volume of media containing 10% serum was added to the cells. Cells were harvested or fixed 48 h after viral transduction.

Immunofluorescence and Microscopy: Cells were seeded onto coverslips and 18 h later were transiently transfected with plasmid. Cells were fixed with 4% paraformaldehyde (VWR/BDH Chemicals, Ottawa, ON, Canada), washed with PBS and then incubated for 1 h at room temperature with PBS containing 5% normal goat serum and 0.3% TX-100 to permeabilize and block samples. Coverslips were incubated overnight in the primary antibody at 4 °C in humid chambers. Washed slides were incubated with a secondary antibody (1:250) for 1 hat RT in the dark. After washing with PBS coverslips were mounted onto slides with VectaShield Antifade Mounting Medium with DAPI (Vector Labs, Burlingame, CA, USA) or ProLong Gold Antifade Mountant (ThermoFisher) and sealed with nail polish. Images were acquired using an epifluorescence microscope (Axio-Observer.D1, Carl Zeiss, Toronto, ON, Canada) equipped with Axiovision 4.8 software (Carl Zeiss). For confocal images and surface rendering, image acquisition was performed on a LSM800 AxioObserver Z1 confocal microscope (Carl Zeiss) using a 100× /1.4 NA objective. The 3D model in Figure 5A was created from Z-stacks acquired with a 0.25 μm step followed by processing with the IMARIS 9.0.2 software (Bitplane, Concord, MA, USA). Image quantification was performed using Photoshop CS6 (Adobe, Ottawa, ON, Canada) or Fiji (https://imagej.net/Fiji).

Senescence-associated (SA)-β-gal staining: Staining was performed as described in [24] to detect senescent cells by Mei Zhang (Ottawa Hospital Research Institute, Ottawa, ON, Canada). Cells were visualized under a phase-contrast microscope and images were acquired using a Zeiss Axiophot microscope with Northern Eclipse software, Version 8.0 (Empix Imaging Inc., Mississauga, ON, Canada).

Statistical Analysis: Statistics were performed using GraphPad Prism 5 (GraphPad, San Diego, CA, USA) and levels of significance indicated in the figure legends.

## 3. Results

### 3.1. MCF-7 Cells Extrude Endogenous SRC-3 through Nuclear “Budding” and Altered SRC-3: CBP Stoichiometry Regulates Nuclear Localization and Expulsion: Many Breast Tumors Express Amplified Levels of SRC-3

**Figure 1:**
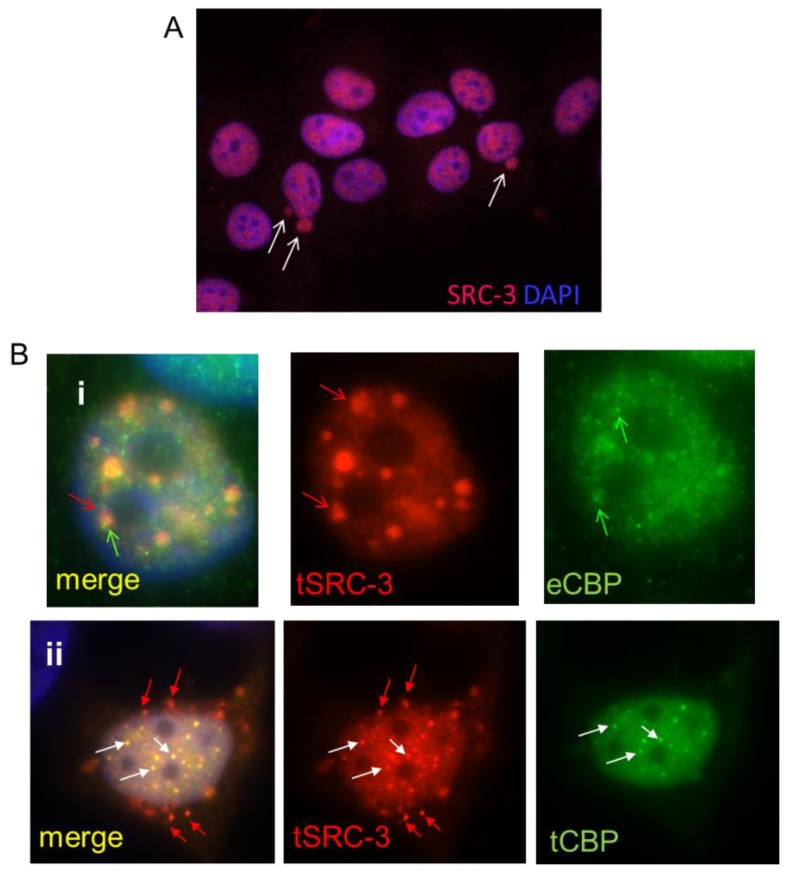
SRC-3 undergoes nuclear extrusion dependent on association with CBP. (**A**) MCF-7 cells cultured as described in Methods were subjected to IF for SRC-3. DNA was visualized with DAPI. Arrows indicate nuclear SRC-3 protrusions (63× magnification). (**B**) MCF-7 cells transfected with SRC-3 (tSRC-3) in the absence (endogenous only- eCBP)(panel i) or presence of cotransfected (tCBP) CBP (panel ii). In panel (i) Z-stack images show SRC-3 aggregates overlapping with diffuse CBP (red arrows). CBP not associated with SRC-3 is indicated by the green arrow. In panel (ii) some aggregates merge as CBP and SRC-3 foci (white arrows), while others near the nuclear periphery contain only SRC-3 (red arrows) (100× magnification).

Among available human breast cancer cell lines, only MCF-7 and BT474 [25] demonstrate SRC-3 gene amplification and express high levels of the protein while the balance of breast cancer cell lines tested (10 ER^+^ and one ER-negative (ER^−^)) have no gene amplification and moderate to low SRC-3 protein expression [26,27]. For this reason, we chose to use MCF-7 cells for these studies. Mechanisms controlling the nuclear content of this IDP outside of transcription-coupled degradation are not known. Since large complexes may undergo nucleo-cytoplasmic transfer through nuclear pore-independent mechanisms we first examined SRC-3 localization by IF of MCF-7 cells. IF of MCF-7 cells for SRC-3 indicated variation in expression across the population and with some cells displaying higher levels than others. Remarkably, a small proportion of cells (approximately 5% of MCF-7 cells) demonstrated large SRC-3 containing vesicle-like budding nuclear protrusions (Figure 1A). Thus SRC-3 appears to undergo nuclear protein control through nuclear budding. We also analyzed SRC-3 by IF in T47-D and ZR-75-1 breast cancer cell lines which express less SRC-3 relative to MCF-7 cells [26,27]. Even with lower SRC-3 protein content, both T47-D and ZR-75-1 lines exhibited nuclear protrusions/blebs containing SRC-3 (Supplementary Figure S1). Analysis of anti-SRC-3 IHC staining of human breast cancer sections from the Protein Atlas collection (www.proteinatlas.org/ENSG00000124151-NCOA3/pathology/breast+cancer) also showed evidence of SRC-3-positive extranuclear budding at a frequency ranging from 2–5% of cells (Supplementary Figure S2). Therefore nuclear clearance of SRC-3 appears to involve a nuclear budding mechanism.

As discussed above SRC-3 binds and synergistically folds with the histone acetyltransferase CBP in the process of initiation of transcription of target genes. If excess SRC-3 exceeds the capacity of CBP, then misfolded SRC-3 might form aggregates and/or require nuclear sequestration or nuclear exclusion. To assess whether overexpressed SRC-3 colocalizes with CBP, we first performed IF after transfection of SRC-3 (tSRC-3) alone. Transfected SRC-3 formed large aggregates, only small regions of which overlap with endogenous CBP (eCBP) (Figure 1, panel i). When both CBP and SRC-3 were overexpressed in the same cell more co-aggregates were observed however SRC-3 aggregates not associated with CBP were perinuclear and underwent nuclear extrusion (Figure 1, panel ii). Thus, excess SRC-3 not associated with CBP forms independent aggregates that are expelled from the nucleus while large nuclear bodies containing CBP and SRC-3 are not, consistent with nuclear retention of folded complexes and expulsion of unfolded SRC-3. This result also indicates a requirement for stoichiometric nuclear expression of SRC-3 folding partners such as CBP, necessary to prevent SRC-3 aggregation.

### 3.2. Overexpressed SRC-3 Induces Cell Cycle Arrest, Senescence, and Apoptosis

**Figure 2:**
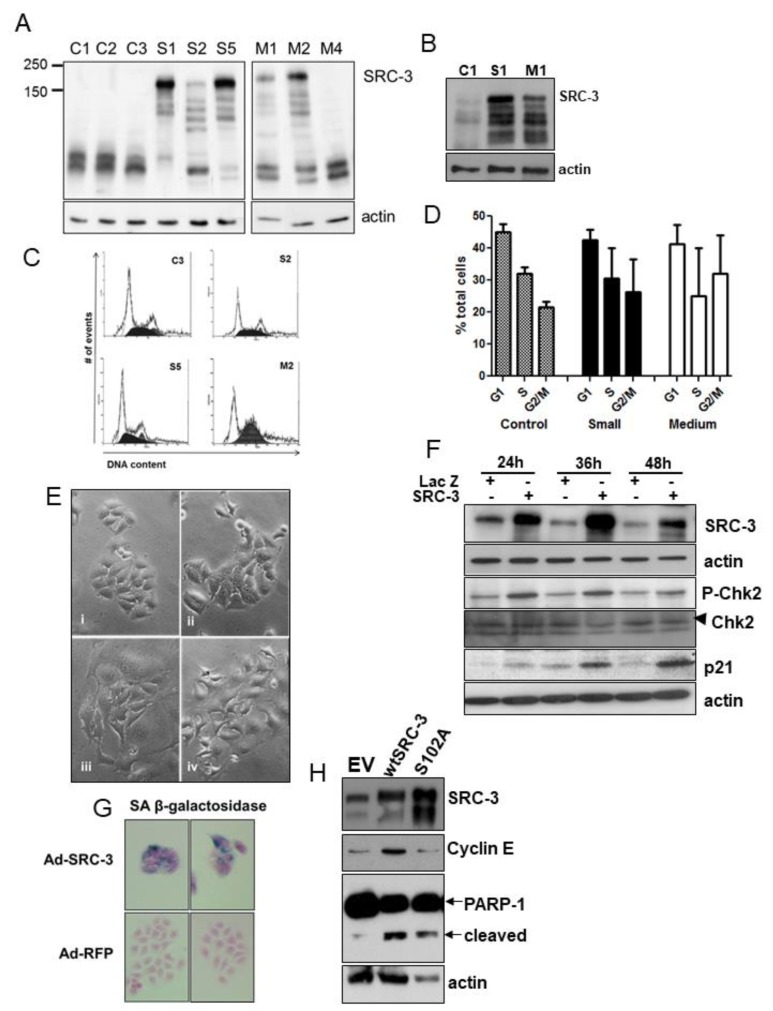
Establishment of SRC-3-overexpressing MCF-7 cells. (**A**) Immunoblot analysis showing SRC-3 protein expression in MCF-7 cells transfected with pCMX-RAC3 (SRC-3) or pcDNA3 and selected for puromycin resistance. SRC-3 stable cells were categorized according to the relative size of the average cell within a colony prior to expansion. Examples of clones that survived at least one passage are shown: control pcDNA3 cells (1,2,3), small-sized SRC-3 transfected clones (S1,2,5) and medium-sized SRC-3 clones (M1,2,4). Large cell clones did not survive passage. (**B**) Immunoblot of original clones showing SRC-3 expression relative to empty vector-transfected control. (**C**) Flow analysis of DNA content derived from the indicated clones. (**D**) DNA content graphs derived from the analysis of several clones. Bars are SD of means from: pcDNA control (*n* = 4), SRC-3 small (*n* = 15), SRC-3 medium (*n* = 4). (**E**) Phase-contrast images of control pcDNA3- and pCMX-RAC3- (SRC-3) transfected MCF-7 cells. Panels are (i) Control clones, (ii) small SRC-3-overexpressing clones, (iii and iv) medium SRC-3-overexpressing clones which were enlarged and flat with abundant cytoplasm. (**F**) Cell lysates harvested after infection at the indicated times after infection with Ad-LacZ or Ad-SRC-3 were probed with antibodies to SRC-3, P-Chk2, Chk2 (denoted by arrowhead), p21 and actin. (**G**) MCF-7 cells infected with the Ad-RFP and Ad-SRC-3 viruses were cultured for 72 h and assayed for senescence-associated (SA) β-galactosidase activity. (**H**) Immunoblot for SRC-3, cyclin E and PARP-1 in MCF-7 cell lysates 72 h post-transfection with empty vector (EV), wtSRC-3 or the stable mutant SRC-3(S102A). Note that although highly expressed relative to wtSRC-3 and to the gel loading control, S102A does not induce transcription of cyclin E. Actin was used as a protein loading control.

Since it appeared that SRC-3 nuclear levels are regulated in part through vesicle-like budding we tested the effect of increasing SRC-3 protein in MCF-7 cells. However, attempts at establishing MCF-7(SRC-3) clones largely failed as cells did not survive the expansion process. We only were able to isolate six clones that survived through limited proliferation expressing varying levels of SRC-3 protein and consisting of small to medium-sized cells (Figure 2A). Relative to MCF-7(SRC-3) clones, on the same blot endogenous 160kDa SRC-3 was not detected at this exposure. Low molecular weight degradation products were clearly visible and are associated with turnover of SRC-3 in conjunction with the estrogen receptor in rapidly dividing cells cultured in estrogenic media [9]. However after passage, small and medium-sized clones expressing SRC-3 grew very slowly (see below) with various levels of SRC-3 degradation. Clone M3 appeared to have ceased expression of SRC-3 possibly due to the formation of senescence-associated heterochromatin (see below). Relative expression of SRC-3 protein in control and transfected colonies isolated at clonal selection is shown in Figure 2B. Previous reports have demonstrated that stable clones of transfected SRC-3 have distinct morphological changes such as increased cell size [28]. Indeed, SRC-3 transfected colonies contained large, flattened, sometimes multi-nucleated cells (Figure 2C). While pcDNA3 control cells (C3) had normal cell cycle phase distributions, most SRC-3 overexpressing clones proliferated very slowly and had aberrant (greater than 2N) DNA content that varied widely (Figure 2D,E), consistent with an aneuploid S/G2/M phase delay or arrest.

Immunoblot of MCF-7 cells transiently infected with adenoviral-SRC-3 (Ad-SRC-3) showed induction of phosphorylated (P)-Chk2, and p21 (Figure 2F). Approximately 80 percent of cells demonstrated senescence-associated(SA) β-galactosidase expression 72hrs post-Ad-SRC-3 infection while none of Ad-LacZ cells expressed this senescence marker (Figure 2G). Transfection of cells with wtSRC-3 or a stable mutant of SRC-3 (S102A) also resulted in substantial cell death as indicated by cleaved PARP-1 (Figure 2H). Thus, increasing SRC-3 protein above endogenous levels is highly detrimental to cell viability.

### 3.3. Ectopically Expressed SRC-3 Protein Forms Nuclear Aggregates

**Figure 3:**
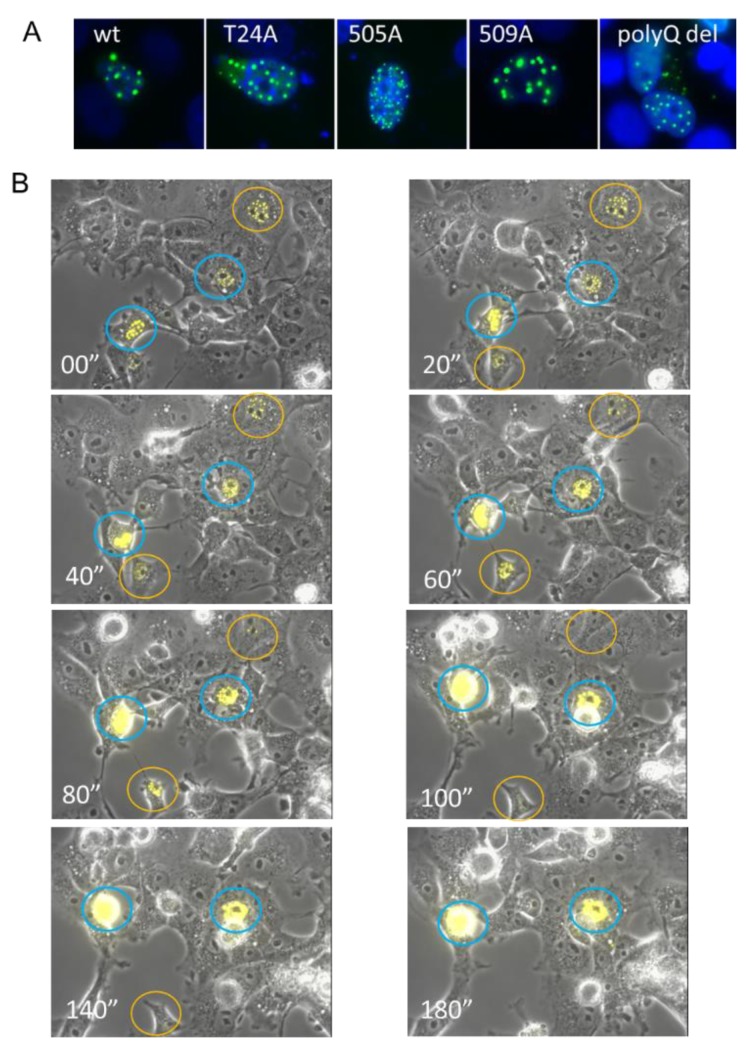
Overexpressed SRC-3 rapidly forms nuclear aggregates. (**A**) GFP imaging of MCF-7 cells 72 h post-transfection with wtSRC-3, three different SRC-3-GFP phospho-mutants, and SRC-3 deleted for the polyQ region (residues 1230–1300). 63× magnification (**B**) Still images from video-microscopy of aggregation of YFP-SRC-3 in transfected cells. MCF-7 cells were transfected with YFP-SRC-3 and microscopy was performed 24 h later on an Axiovert 200M inverted fluorescent microscope (Carl Zeiss, Toronto, ON, Canada) for a total of 24 using Axiovision 4.8 acquisition software (Carl Zeiss). Images were acquired using a 10× objective (EC Plan-Neofluar) with a side-mounted AxiocamHRm camera (Carl Zeiss). YFP was excited using the Colibri LED illumination system (LEDmodule 505nm, Carl Zeiss) and detected using the 46HEYFP filter (Carl Zeiss). Exposure times were 1 ms (brightfield/phase contrast) and 100ms (YFP) at 10 min intervals for 24 h and compiled into video files using Axiovision 4.8 software (Carl Zeiss). 20 min intervals are shown. Cells circled in blue showed continuous accumulation of SRC-3 while cells circled in orange appeared to resolve aggregates.

To understand the mechanism of SRC-3-induced cytotoxicity/senescence we performed IF. Strikingly, transiently transfected SRC-3 was either homogeneously distributed in the nucleus or formed solid or ring shaped-nuclear aggregates (Figure 3A). Alanine substitution mutants of SRC-3 at previously identified phosphoserines were all able to form aggregates as was a mutant deleted of the polyQ region [9].

Previous studies of a GFP-tagged disordered nuclear protein called GFP170* showed that small aggregates of GFP170 * form at or adjacent to PML bodies and then move toward each other and fuse to form larger aggregates accompanied by spatial rearrangements of the PML bodies [29]. Live cell imaging of SRC-3-YFP-transfected cells shows that aggregates SRC-3 foci formed rapidly (within 3 h) from the first appearance of puncta. In some cells, they coalesced and resulted in cell death (circled in blue) while in other cells they reached a maximum size then began to dissipate (circled in orange) (Figure 3B).

### 3.4. SRC-3 Overexpression Does Not Affect the Proteasome but Induces Autophagy

**Figure 4:**
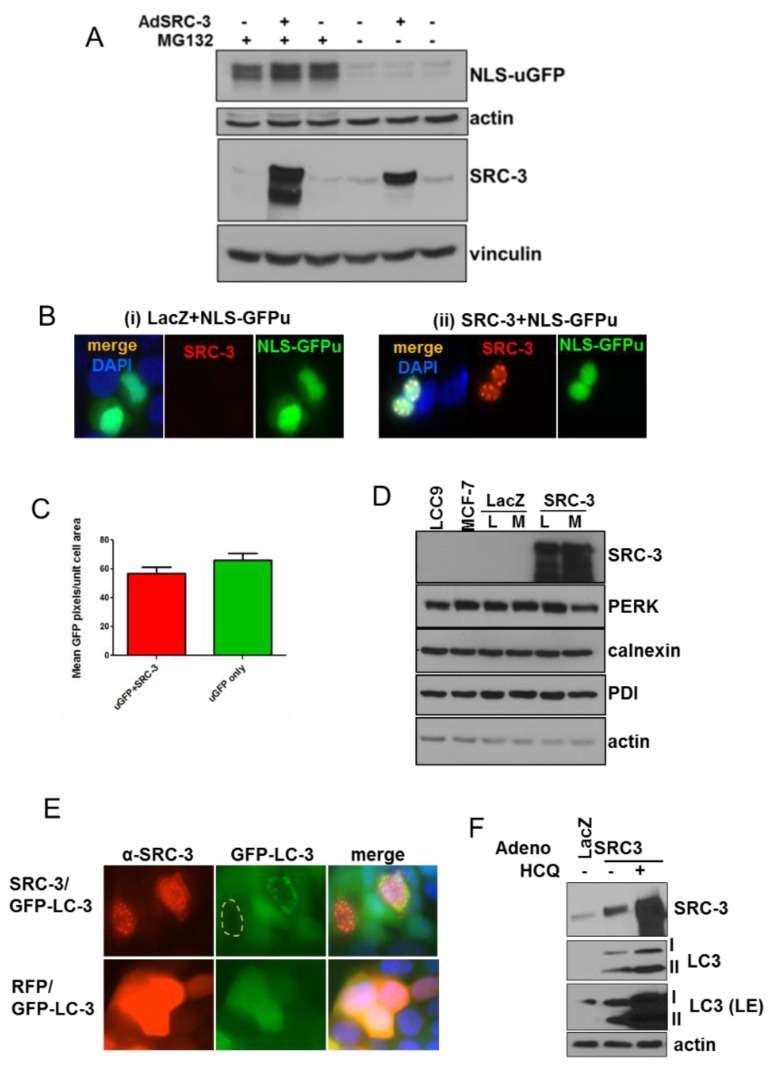
SRC-3 overexpression induces autophagy but does not affect the proteasome. (**A**) MCF-7 cells stably transfected with a nuclear-targeted GFP (NLS-GFPu) were mock-infected, or infected with Ad-SRC-3 or Ad-Lac-Z. Cells harvested 72 h after infection with or without 10 μM MG132 for the final 3 h of culture. Immunoblots were probed with anti-GFP and anti-SRC-3. Anti-actin reactivity was used as a control for protein loading on anti-GFP-probed blots. E-cadherin or vinculin were used as loading controls for blots probed with anti-SRC-3. (**B**) Cells infected as in **A** were seeded on coverslips and IF was performed after 72 h. GFP (green), anti-SRC-3/CY5 (red) and DAPI fluorescence are shown (63× magnification). (**C**) Graph depicting average of uGFP pixels/nuclear area within nuclei of MCF-7 cells described as above in **A**. Bars are S.E.M. (**D**) Immunoblots to detect the activation of the unfolded-protein response in LCC9 (L) (a tamoxifen-resistant MCF-7 derivative [30]) and MCF-7 (M) cells uninfected or infected with adenoviral (Ad)-LacZ or Ad-SRC-3. Actin reactivity serves as a protein loading control. (**E**) MCF-7 cells stably expressing LC3-EGFP were infected with Ad-SRC-3 or Ad-RFP. Anti-SRC-3 and GFP-LC-3 were detected by epifluorescence microscopy (63× magnification). (**F**) MCF-7 cells were infected with Ad-Lac Z or Ad-SRC-3 and treated with a vehicle or 20 μM HCQ for 48 h. Lysates were immunoblotted for SRC-3, LC3 or actin, (LE denotes long exposure).

Cytoplasmic aggresomes include ubiquitin and proteasomal subunits [29] that eventually overwhelm the proteasome [17]. To assess whether nuclear SRC-3 overexpression affected global proteasome function, we stably transfected MCF-7 cells with an unstable variant of GFP tagged with a nuclear localization motif (NLS-GFPu) [19] then infected these cells with adenovirus expressing SRC-3(Ad-SRC-3). Immunoblot showed the presence of NLS-GFPu in cells treated with MG132, a proteasome inhibitor, compared with vehicle-treated cells while no protein was detected following either Ad-SRC-3 or Ad-LacZ infection (Figure 4A). To assess proteasome inhibition by SRC-3 overexpression in individual cells we performed IF for SRC-3 and GFP in Ad-LacZ and Ad-SRC-3 infected cells transfected with NLS-GFPu. GFP-fluorescence in Figure 4B shows that cells co-expressing the GFPu and transfected SRC-3 express similar levels as GFPu in control/LacZ-transfected cells quantified in Figure 4C. Thus SRC-3 nuclear aggregates do not block the 26S proteasome. Moreover, overexpression of SRC-3 did not induce markers of the unfolded protein response (UPR) in either MCF-7 cells or LCC9 tamoxifen-resistant derivative cells [30] including the absence of a P-PERK shift associated with inhibition of translation, induction of the UPR genes CHOP (cell cycle arrest), PDI (protein folding) [31] (and references therein) or calnexin which controls protein transport for ER-associated degradation [32] (Figure 4D).

Since nuclear SRC-3 expression had no effect on proteasome activity or induce the UPR, we next tested whether it induced the autophagic degradation pathway. Adenoviral transduction of SRC-3 in MCF-7 cells stably expressing eGFP-LC3 resulted in perinuclear formation of autophagic vesicles in many cells overexpressing SRC-3 (Figure 4E). Furthermore, SRC-3 transduced MCF-7 cells displayed increased formation of cleaved/phosphatidyl-ethanolamine conjugated LC3 (LC3-II) indicating the induction of autophagy (Figure 4F). Transduced SRC-3 was further stabilized by the autophagy inhibitor, hydroxychloroquine (HCQ). Together these data indicate that overexpressed SRC-3 protein is proteotoxic and can form nuclear aggregates that induce an autophagic response.

### 3.5. SRC-3 Nuclear Aggregates Are Proximal to Microtubules and Overexpression Disrupts Both Mitotic Microtubule Dynamics and the Nuclear Membrane

**Figure 5:**
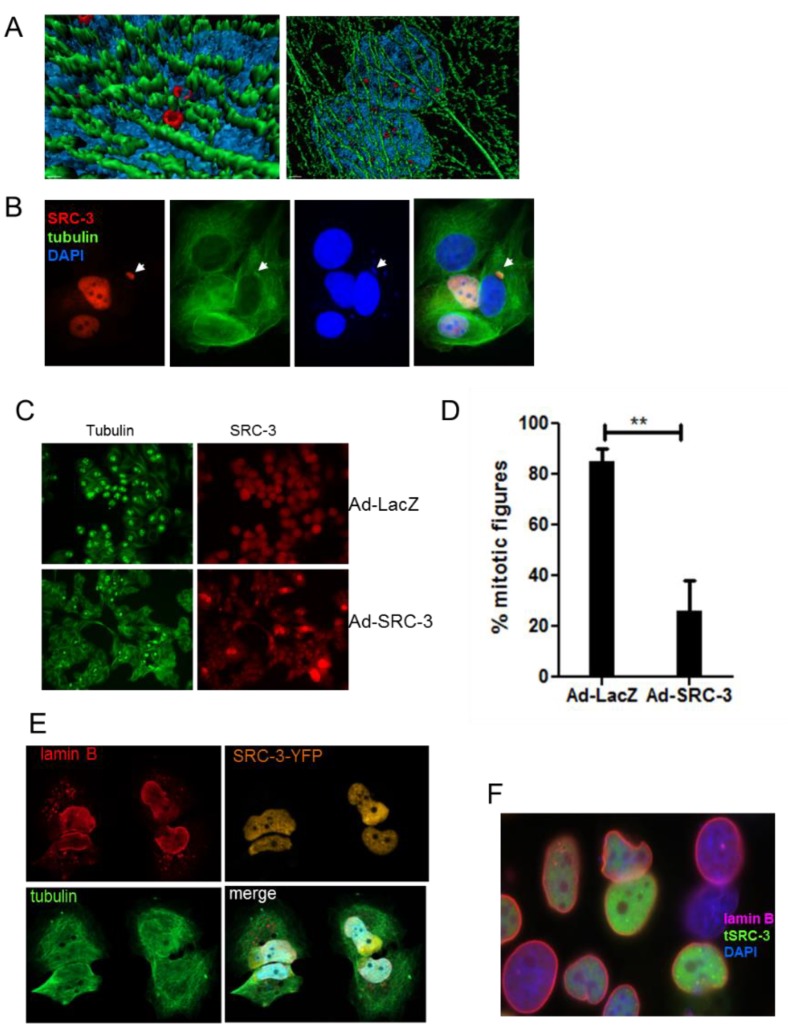
SRC-3 aggregates are associated with microtubules, redistribute PML and SUMO and compromise nuclear membrane integrity. (**A**) 3D deconvoluted model constructed as described in Materials and Methods using Z-stacks acquired with confocal microscopy from SRC-3-transfected cells and immunostained for SRC-3 (red) and β-tubulin (green) and imaged. Blue is DAPI (100× magnification). (**B**) Immunofluorescence of β-tubulin (green) and SRC-3 (red) showing regions of increased perinuclear tubulin density in SRC-3 overexpressing cells. Nuclear protrusion of SRC-3 and DNA (blue) are indicated with arrows (63× magnification). (**C**) SRC-3 expression interferes with mitotic microtubule dynamics. MCF-7 cells were infected with Ad-LacZ or Ad-SRC-3 on coverslips and 48 h later were treated with taxol for 18hrs and IF performed to detect mitotic microtubules and SRC-3 (63× magnification). (**D**) Cells treated in **C** were enumerated for the presence of mitotic figures. Bars are S.D., ** *p* < 0.01. (**E**) Z-stack images of cells transfected with SRC-3-YFP and immunostained for lamin B1 (red) and tubulin (green) immunofluorescence showing nuclear membrane distortion, and degradation associated with cytoplasmic SRC-3 aggregates (100× magnification). (**F**) IF of lamin B1 in SRC-3-GFP-transfected cells showing cells with discontinuous nuclear membranes, lamin B1 fragments, and distortions of nuclear shape.

To further characterize the striking cytotoxicity of excess SRC-3 in MCF-7 cells we examined mechanisms associated with protein aggregate formation and resolution. Small aggregates of cytoplasmic misfolded proteins form and are rapidly transported toward the microtubule (MT)-organizing center, where they coalesce to form aggresomes [29]. In Figure 5A, deconvoluted 3-D images showed that SRC-3 nuclear aggregates were invariably closely associated with perinuclear microtubules. To investigate whether SRC-3 overexpression would interfere with mitotic microtubule dynamics we treated cells with 10 nM taxol for 18–24 h post-infection with Ad-LacZ or Ad-SRC-3 and evaluated mitotic figures. Figure 5B shows that overexpressed homogeneously distributed SRC-3 disrupted the microtubular network resulting in a perinuclear concentration of tubulin in cells without or possibly prior to aggregate formation. In some cells tubulin was colocalized with SRC-3-containing nuclear cap-like structures in cells transfected with SRC-3 in the absence of aggregates. MCF-7 clones expressing SRC-3 had aberrant DNA content profiles which could be a consequence of microtubular dysfunction. The results in Figure 5C demonstrate a significant reduction in metaphase cells as determined by metaphase microtubule structures in cells infected with Ad-SRC-3 but not Ad-LacZ following a 12 h exposure to taxol. The enumeration of mitotic figures in each condition is shown in Figure 5D. Degradation of the nuclear lamina by autophagy was initially discovered as a response to oncogenic stress–a process termed “nucleophagy” [33]. Since SRC-3 overexpression resulted in nuclear membrane perturbation we acquired Z-stack images of YFP-tagged SRC-3, tubulin and lamin B1 in cells overexpressing SRC-3. Figure 5E shows the distortion and disruption of nuclei accompanied the release of lamin B1 into the cytoplasm. A variety of lamin B1 nuclear disruptions patternswere observed accompanying increased SRC-3 expression. In Figure 5F a misshapen nucleus can be seen as well as regions of discontinuous lamin B1 IF in all cells where transfected SRC-3 is expressed.

Thus, the senescence and cytotoxicity induced by increasing SRC-3 protein levels in MCF-7 cells most likely derive from the effect of nuclear aggregate formation on microtubule function and nucleophagy-mediated cell death.

### 3.6. SRC-3 Overexpression Redistributes PML Bodies and Induces PML

**Figure 6:**
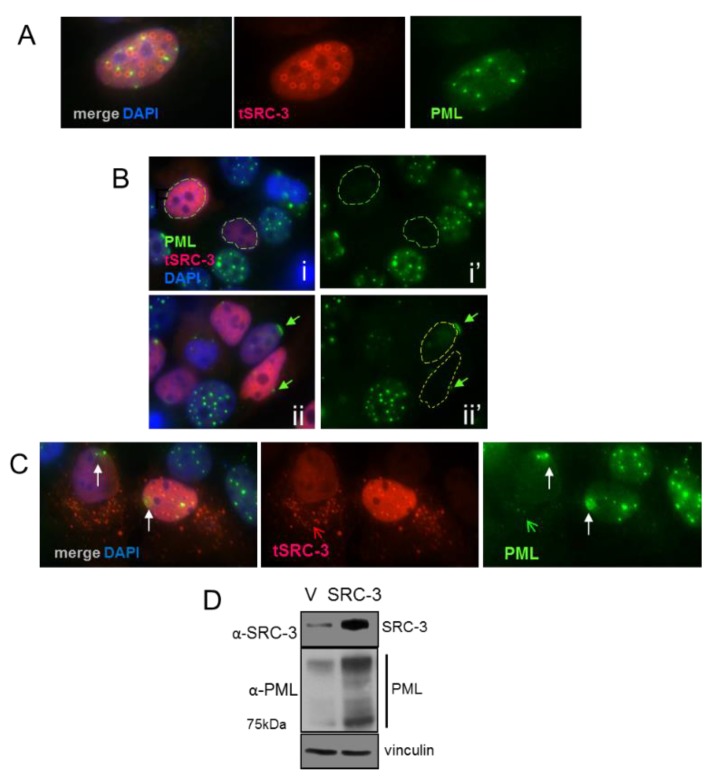
Effects of increased SRC-3 expression on PML: (**A**) IF of PML and SRC-3 in SRC-3 transfected cells shows ring-like aggregates of SRC-3 adjacent to PML bodies. (**B**) Dispersion of PML bodies (circled nuclei in panels i and ii’) in MCF-7 cells containing varying levels of homogenously distributed SRC-3. Nuclear membrane accumulation into caps of PML was observed in a subset of SRC-3 transfected cells (arrow in panels ii and ii’) (63× magnification). (**C**) Diffuse accumulations of PML form near the nuclear membrane. Cytoplasmic SRC-3(red open arrow) and PML (green open arrow). (**D**) Immunoblot of PML 96hrs following SRC-3 transfection. “V” denotes empty vector control. Vinculin was used as a protein loading control.

The synthetic disordered protein GFP170* forms aggregates that coalesce at PML bodies resulting in the redistribution of PML [29]. To determine the effect of SRC-3 on PML bodies we performed IF of MCF-7 cells transiently expressing varying levels of transfected SRC-3. Similar to GFP170*, Figure 6A shows ring-like SRC-3 aggregates that form adjacent to PML bodies. In cells expressing homogenously distributed SRC-3, PML bodies were either undetectable (Figure 6B (i,i’) or PML was focused on nuclear membrane caps (Figure 6B (ii,ii’) (arrow)). In other examples, small SRC-3 aggregates were released from the nucleus along with PML. In the absence of SRC-3 aggregate formation PML formed diffuse masses near the nuclear membrane (Figure 6C (arrows)). Thus, aggregate formation and homogenous increases in SRC-3 have distinctive effects on PML. Oncogenic ras [34] and myc [35] are IDPs that induce PML upon overexpression resulting in senescence. Consistent with the induction of senescence by SRC-3 (see Figure 1F), a strong increase in PML protein was observed 96hrs following transfection of SRC-3 (Figure 6D).

### 3.7. Increased SRC-3 Protein Level Impacts the SUMO Pathway

**Figure 7:**
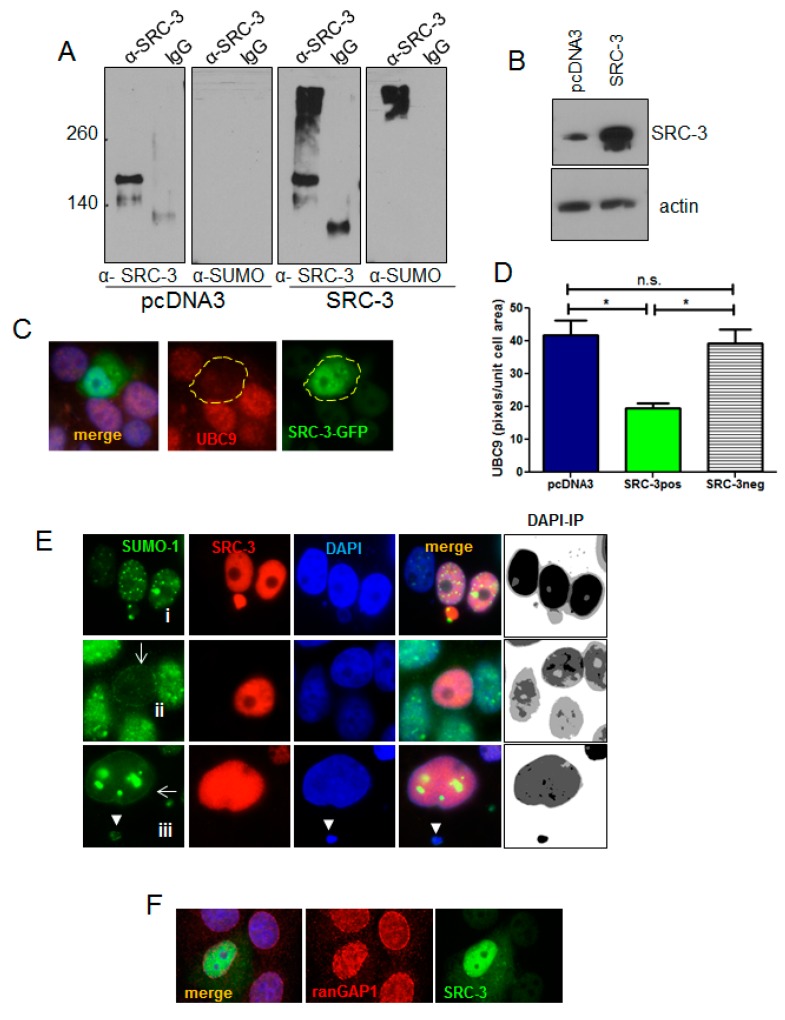
Overexpressed SRC-3 is SUMOylated and disrupts the SUMO pathway. (**A**) Immunoprecipitation of protein from control (pcDNA3) or SRC-3 transfected MCF-7 cells using anti-SRC-3 or non-immune IgG was immunoblotted with anti-SUMO and anti-SRC-3. (**B**) SRC-3 immunoblot of input cell lysates in A as indicated. (**C**) MCF-7 cells were transfected with SRC-3-GFP then IF performed for UBC9. A representative cell expressing transfected SRC-3 (SRC-3-GFP^+^) is shown (63× magnification). (**D**) Graph depicting mean of UBC9 pixels/nuclear area within cells transfected with SRC-3-GFP (*n* = 12 GFP^pos^ cells and *n* = 20 GFP^neg^ cells as shown) or transfected with pcDNA3 (*n* = 25 cells) ± S.E. * *p* < 0.05 (paired t-test). (**E**) Examples of SUMO IF in SRC-3 overexpressing MCF-7 cells. Panel (i): SUMO-1 puncta in the nucleus and associated with an SRC-3/chromatin nuclear protrusion. Panel (ii): Nuclear membrane-associated SUMO-1 with loss of nuclear puncta in an SRC-3 overexpressing cell. Panel (iii): MCF-7 cell with SUMO-1 aggregates and SUMO-1 distribution at the nuclear membrane. Note the absence of detectable nucleoli. Images on the right of each panel represent inverted and posturized (IP) DAPI channel images (Photoshop CS6) to visualize extranuclear DNA. (**F**) RanGAP1 IF (red) in MCF-7 cells transfected with SRC-3 (green) shows redistribution of ranGAP1 at the nuclear periphery. DNA is stained with DAPI (blue) (E and F, 63× magnification).

SUMOylation has a critical role in proteostasis and regulates nuclear protein aggregation in neurodegenerative diseases [36]. SUMOylation is also involved in the formation of PML nuclear bodies [37,38] which were strongly reduced in SRC-3 overexpressing cells. PML bodies are themselves the major sites of SUMOylation of PML client proteins [39]. We, therefore, tested the involvement of the SUMO pathway following overexpression of SRC-3. Immunoprecipitated SRC-3 from SRC-3 transfected cells appears as a very high molecular weight smear in addition to the 160kDa band consistent with poorly detergent soluble SUMO-SRC-3 aggregates (Figure 7A,B). UBC9 is the only known E2 SUMO-conjugating enzyme [40]. Therefore, we tested the effects of SRC-3-GFP overexpression on UBC9 protein levels by IF. Figure 7C shows a representative SRC-3-GFP-positive cell in which UBC9 IF staining intensity was markedly reduced. Average IF pixel values quantified for UBC9 (CY3) in SRC-3-GFP-positive cells and GFP-negative cells are graphed (Figure 7D) indicating a significant decrease in detectable UBC9 in SRC-3 overexpressing cells. Thus, ectopic SRC-3 protein is SUMOylated and accompanied by a reduction in detectable UBC9. Since transfected SRC-3 was SUMOylated and redistributed PML, we next determined its effects on the cellular distribution of SUMO-1by IF. The panels in Figure 7E depict the various structural and localization changes that occur in SRC-3 overexpressing cells. Figure 7E, panel i shows discrete SUMO-1 foci in an overexpressing cell. One cell shows an SRC-3 containing protrusion into the cytoplasm in association with SUMO-1. In some expressing cells SUMO-1 was restricted to the nuclear periphery (Figure 7E, panels ii,iii). In Figure 7E, panel iii the enlarged cell shows large foci of SUMO-1. Interestingly, nucleoli numbers were reduced to one or were undetectable as assessed using DAPI- a phenomenon previously reported in cells lacking UBC9 [41].

Importantly, SUMOylation of nuclear membrane laminA/C has been shown to be required for the interaction between LC3 and lamin A/C to facilitate the process of nucleophagy in response to DNA damage [42]. Similar to Figure 4B showing tubulin-associated extranuclear chromatin, cytoplasmic chromatin surrounded by SUMO-1 is also present in panel iii (arrowhead).

The GTPase activating protein, ranGAP1, is a key enzyme in the ran-controlled receptor-mediated nuclear pore transport system [43]. SUMOylation of ranGAP1 is essential for nuclear membrane localization and it can be detected using anti-SUMO-1 ([44] and references therein). IF for ran-GAP1 in SRC-3 transfected cells showed disruption in the normal localization at the nuclear membrane (Figure 7F).

### 3.8. Pharmacologic Stabilization of SRC-3 Recapitulates Effects of Overexpression

In the experiments above we noted that overexpression of levels of SRC-3 that do not result in aggregation was sufficient to result in nucleophagic-like expulsion of SRC-3 from the nucleus. We reasoned that clinically relevant treatment modalities that are associated with senescence induction and or proteasome/autophagy inhibition have the potential to increase SRC-3 levels [45] and may, therefore, result in similar responses similar to ectopic expression.

**Figure 8:**
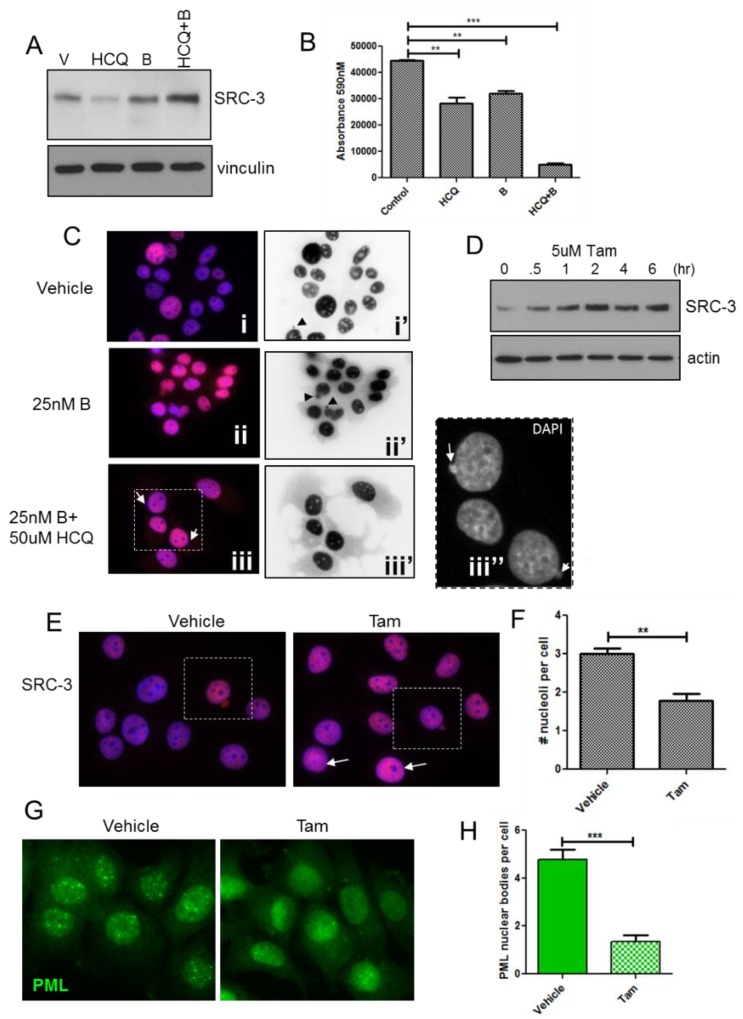
Pharmacological protein stabilization results in strong increases in cytoplasmic SRC-3 and correlates with growth inhibition. (**A**) Immunoblot of SRC-3 after a 48hr treatment with vehicle, HCQ, B or HCQ+B. Vinculin immunoreactivity was used is a protein loading control. (**B**) MCF-7 cells were treated as indicated for 96 h and viability assessed by Alamar blue assay. Cells were assayed in quadruplicate. Bars are mean ± S.D. ** *p* < 0.01, *** *p* < 0.01 (One-way ANOVA). (**C**) IF of SRC-3 in MCF-7 cells treated with vehicle or B or HCQ+B for 48hrs. Panels (i, ii, iii) merged DAPI (blue)/anti-SRC-3 (red) pseudocolored IF, panels (i’,ii’,iii’) anti-SRC-3 epifluorescence only (identical exposure images inverted in Photoshop CS6). Panel iii’’ represents DAPI only (grayscale) enlargement of the boxed area in (iii). Arrows point to extranuclear chromatin. All images 63× magnification. (**D**) Immunoblot for SRC-3 in MCF-7 lysates from cells treated for the indicated times in 5 μM tamoxifen. Vinculin was used as a protein loading control. (**E**), IF for SRC-3 in cells treated with vehicle or 5 μM Tam for 3 h. Cells with SRC-3 nuclear protrusions are boxed. Cells with reduced nucleoli are indicated with closed arrows. (**F**), Nucleolar regions were enumerated from DAPI-stained images in cultures treated as in (**E**). Bars are S.E.M. ** *p* < 0.01. (**G**), IF to detect PML protein in MCF-7 cells treated as in (**E**). (**H**) Enumeration of discrete PML bodies in cells treated as in (**E**). Bars are S.E.M. *** *p* < 0.001(paired t-test).

Proteasome and autophagy inhibitors have been utilized often in combination with other therapeutics, in vitro and clinically to induce cytotoxicity in breast cancer cells and tumors [46,47,48] albeit sometimes with limited success [49]. We tested the effects of proteasome and autophagy inhibition on levels and localization of SRC-3. Immunoblot analysis in Figure 8A shows that the proteasome inhibitor, bortezomib (B), only slightly increased SRC-3 in MCF-7 cells, possibly due to increased autophagic clearance as described elsewhere [50]. Unexpectedly, we found that HCQtreatment alone strongly reduced SRC-3 protein levels, a finding which may reflect increased proteasomal activity when the autophagic removal of proteasome components is inhibited [51]. The combination of B with HCQ increased the levels of SRC-3 protein and treatment for, 72 h with this combination also resulted in a significantly higher level of cytotoxicity compared to either agent alone (Figure 8B). To assess the effects of the B+HCQ treatment on SRC-3 at the cellular level we performed IF. Figure 8C, panel (i) depicts SRC-3 IF in cells treated with vehicle and shows again that a subset of cells exhibit nuclear protrusions containing SRC-3 although even in high contrast epifluorescence images (panel i’) virtually no SRC-3 was detected in the cytoplasm. In contrast a 72-h exposure to B or B+HCQ strongly increased the level of SRC-3 in the cytoplasm (Figure 8C, panels ii/ii’ and iii/iii’). Moreover, extrusion of chromatin into the cytoplasm was also observed in combination-treated cells (Figure 8C, panel iii’’). Thus, inhibition of the proteasome results in aberrant transfer of endogenous SRC-3 to the cytoplasm in approximately 2% of the MCF-7 cells examined. While overall protein stability and downstream effects are also factors in pharmacologic-mediated cytotoxicity of B+HCQ treatment, the presence of SRC-3 within nuclear protrusions and increased cytoplasmic localization demonstrates that SRC-3 is an important substrate for this form of nucleophagy-like egress in response to protein stabilization.

Tamoxifen (Tam) is an important therapeutic in ER+ breast cancer and has also been shown to stabilize SRC-3 protein [52] while promoting senescence in ER+ breast cancer cells [53]. The immunoblot in Figure 8D shows that SRC-3 levels are substantially increased in MCF-7 cells treated with 5 μM Tam within 2 h. Consistent with increased SRC-3 protein levels, Figure 8E shows enhanced SRC-3 IF in Tam-treated MCF-7 cells vs. control following a 3hr exposure to the drug. A cell shown in vehicle-treated cultures appeared to have higher SRC-3 expression relative to surrounding cells and both this cell and cells within the Tam-treated cultures exhibited nuclear SRC-3 protrusions typical of SRC-3 transfected cells. Notably, high expression of SRC-3 in Tam-treated cells was also associated with reduced nucleoli (arrows) as we observed in SRC-3 transfected cells. Similar to cells transfected with SRC-3 (Figure 7), numbers of nucleoli were significantly reduced after the 3 h exposure to Tam (Figure 8F). Since transfected SRC-3 resulted in dispersion of PML nuclear bodies we performed IF for PML in Tam-treated cells. In Figure 8G Tam reduced the detection of discrete PML bodies compared to control. Quantification of PML bodies showed that Tam treatment resulted in a significant decrease compared to vehicle (Figure 8H). Thus, drug-induced stabilization of SRC-3 has similar effects as SRC-3 transfection.

## 4. Discussion

The AIB1 gene is amplified in approximately 5–10 percent of breast cancers while high expression is present in more than 60 percent of primary tumors, typically associated with ER^+^ tumors [54]. SRC-3 is known to be tightly regulated by phosphorylation, dephosphorylation and SUMOylation events that affect both transcriptional activity and post-transcriptional degradation [9]. Since SRC-3 is highly disordered in the absence of folding with CBP [13], rigorous control of unbound protein levels is necessary to avoid potential proteotoxicity. Misfolded nuclear proteins are sequestered at PML bodies and SUMOylated. Indeed, SUMOylation of SRC-3 is associated with loss of SRC-3 coactivator function [11]. Thus SRC-3 may be SUMOylated in the absence of transcriptional activation to facilitate sequestration of unfolded protein. Consistent with this prediction, transfected SRC-3 was SUMOylated and often formed aggregates in MCF-7 cells consistent with previous reports that transfected SRC-3 appears as discrete foci in both HeLa cells [55] and an ER-negative breast cancer cell line [56].

Although it is unclear whether nucleophagy is a normal homeostatic process in eukaryotes [57] there is evidence that it is employed for the clearance of nuclear waste [58]. Interestingly, our finding that SRC-3 nuclear protrusions can be seen even in untreated MCF-7 cells and other breast cancer cell lines and tumors which may be indicative a form of nucleophagy or nuclear “budding” which contributes to the regulation of SRC-3 protein levels.

This mechanism may be particularly important for cancer cells that contain high levels of unfolded transcription factors such as SRC-3 and utilized under conditions where folding is reduced either due to stoichiometric imbalances or inhibition of transcription-coupled degradation. Nuclear budding has been proposed as an alternative mechanism of nuclear protein quality control analogous to Herpesvirus egress. This process involves budding from the nuclear membrane of aggregates or complexes too large for the nuclear pore in order to access the cytoplasmic autophagy machinery [59]. A similar process is utilized in yeast called “piecemeal microautophagy” [60].

Overexpression of SRC-3 can result in SUMOylation of misfolded protein within PML bodies resulting in SUMO-targeted ubiquitination and degradation. However, similar to nucleophagy, nuclear budding is proposed to transport large aggregates destined for autophagic degradation out of the nucleus independent of nuclear pores [61]. We speculate that nuclear budding/piecemeal microautophagy may be utilized for homeostatic removal of SRC-3 from the nucleus while increasing expression induces nucleophagy based on the various cellular phenotypes we observed. For example, nuclear oncogene-induced senescence has been associated with nuclear membrane lamin B1 autophagy [33] while SUMOylation of nuclear lamins occurs during DNA damage-induced nucleophagy [42]. Thus, lamin B1/RanGAP disruption and redistribution of SUMO to the nuclear lamina in SRC-3 overexpressing cells together provide evidence for nucleophagy which may result in gross nuclear disruption and cell death or controlled budding of DNA/SRC-3/tubulin nuclear protrusions (Figure 5B).

We found that overexpression of SRC-3 also altered PML/SUMO localization and disrupted the localization of SUMO-RanGAP1 consistent with perturbation of the SUMO pathway. Similar to other disordered proteins, SRC-3 aggregates accumulate adjacent to PML bodies. However, in the absence of aggregate formation, PML bodies dispersed in response to overexpression of SRC-3. These observations parallel that of cells lacking SUMO-1 which results in aberrant nuclear pore localization of RanGAP1 and dispersion of PML bodies [62]. We speculate that high expression of unfolded transcription factors could contribute to the reduced numbers of PML bodies typical of transformed cells relative to normal cells. PML protein sometimes coalesced and accumulated at the nuclear membrane in some cells in cap-like formations in response to increased levels of SRC-3. Interestingly PML-II can displace lamins from the nuclear membrane [63] which was clearly evident in SRC-3-transfected cells (Figure 5E,F) accompanied by the release of small SRC-3 foci. It is possible that PML may participate in the process of nucleophagy induced by IDPs by contributing to the disassembly of lamins in the nuclear membrane to allow access to the autophagosome.

SUMO E2 enzyme phosphorylation by CDK1/cyclin B during mitosis increases its overall activity [64,65] when SUMOylation events are necessary for proper chromosome segregation [41,66,67]. Thus, the perturbation of the SUMO pathway after SRC-3 overexpression may have contributed to the aneuploidy we observed in MCF-7(SRC-3) colonies as could direct effects on microtubule function. The formation of cytoplasmic “aggresomes” [68] requires microtubules and dynein-based transport to coalesce aggregates. Remarkably, nuclear SRC-3 aggregates in MCF-7 cells were invariably juxtaposed with microtubules. How microtubules might affect nuclear protein aggregation is not clear. Components of the linker of nucleoskeleton and cytoskeleton (LINC) complexes spanning the inner and outer nuclear membranes interact with perinuclear microtubules that exert force on the nucleus through microtubule motors [69,70]. LINC components include nuclear envelope nesprins, which connect the nuclear lamina to the cytoskeleton and molecular motors [71]. Based on the proximity of SRC-3 aggregates to microtubules it is possible that these structures could form organizing centers for aggregation of unfolded SUMOylated nuclear proteins.

Stress on protein quality control mechanisms may ultimately participate in senescence induction when SRC-3 and other nuclear IDPs are stabilized by proteasome/autophagy inhibitors or Tam. In this regard, Tam treatment strongly reduced PML bodies which are dependent on SUMOylation of PML [72] suggesting diversion of SUMO from PML to SRC-3 and possibly other proteins. In the future, it will be of interest to determine if endogenous amplified expression of SRC-3 and other oncogenic IDPs are potential liabilities of cancer cells that can serve as markers of sensitivity to agents that stabilize these proteins or interfere with the processing of IDPs by blocking SUMOylation.

## Supplementary Materials

The following are available online at https://www.mdpi.com/2073-4409/8/10/1278/s1. Figure S1: Immunofluorescence demonstrates discrete SRC-3 cytoplasmic blebs in ER+ breasr cancer cells. Figure S2: Examples of anti-SRC-3 positive extranuclear budding structures in IHC of human breast cancers expressing high levels of SRC-3 indicated by arrows.
