# Supplementary material for: Intrinsically Disordered SRC-3/AIB1 Protein Undergoes Homeostatic Nuclear Extrusion by Nuclear Budding While Ectopic Expression Induces Nucleophagy
Source: Cells. 2019 Oct 19;8(10):1278. doi: 10.3390/cells8101278 (PMC6830083; doi:10.3390/cells8101278)
Supplement: Supplementary file 1 [file cells-08-01278-s001.pdf]

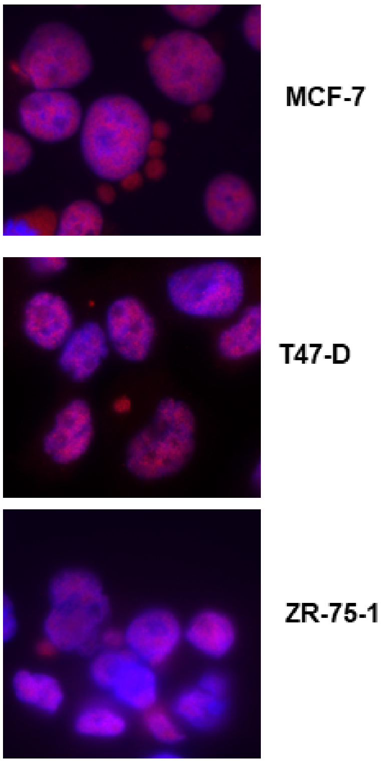

Supplementary Figure 1: Immunofluorescence demonstrates discrete SRC-3 cytoplasmic blebs in ER+ breast cancer cells. In these examples multiple juxtanuclear blebs are seen in MCF-7 cells while fewer T47-D and ZR-75-1 cells displayed blebbing (with a frequency of 1 bleb per positive cell) . IF intensity was enhanced in T47-D and ZR-75-1 relative to MCF-7 cells to better visualize SRC-3.

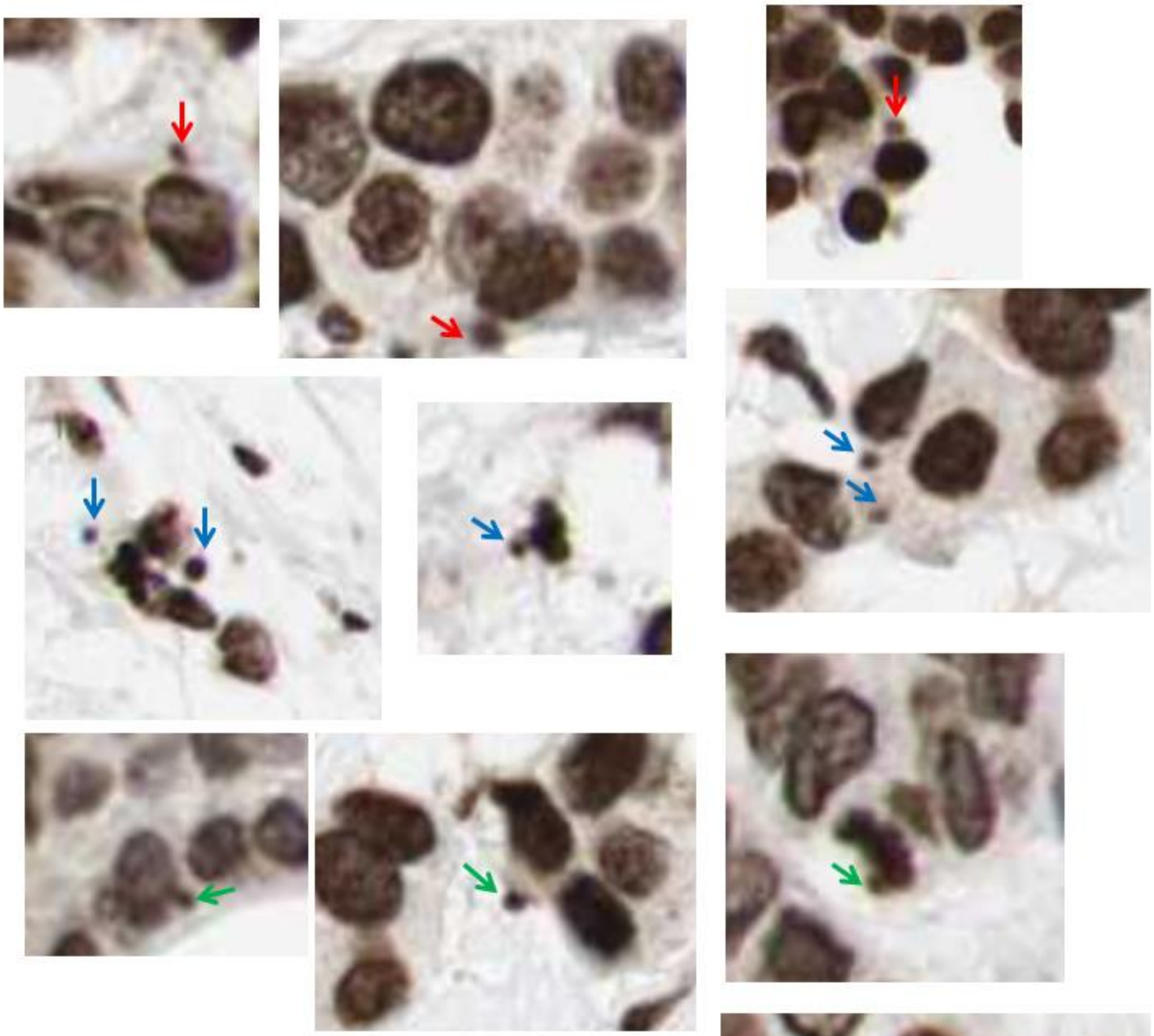

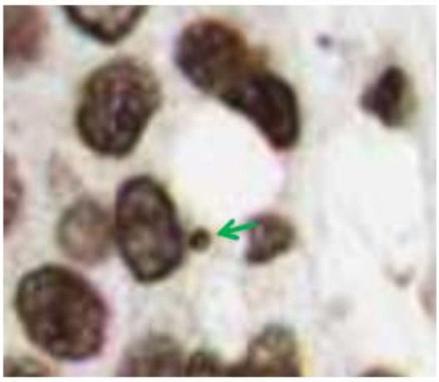

Supplementary Figure 1: Examples of anti-SRC-3 positive extranuclear budding structures in IHC of human breast cancers expressing high levels of SRC-3 indicated by arrows.
Red arrows; id:1882 (medium staining/lobular ca)
Blue arrows; id:3257 (high staining/ductal ca)
Green arrows; id:2428 (high staining/ductal ca)
